# E-cigarettes and Vaping, Product-use Associated Lung Injury: A Case Series of Adolescents

**DOI:** 10.5811/cpcem.2020.10.48707

**Published:** 2021-01-16

**Authors:** Abdullah Khan, Karli Parlette, Heather M. Kuntz

**Affiliations:** Loma Linda University, Department of Emergency Medicine, Loma Linda, California

**Keywords:** Electronic cigarettes, and vaping, product-use associated lung injury, adolescents, emergency department

## Abstract

**Introduction:**

Lung injury associated with the use of electronic cigarettes and vaping (EVALI) was first identified in 2019. Since then, clusters of cases have been reported in the literature. Our aim was to describe the clinical presentation of adolescents with EVALI in the emergency department and their clinical outcomes.

**Case Series:**

In our case series, we identified seven adolescents diagnosed with EVALI. We describe their signs and symptoms on presentation to the emergency department and their clinical course. The most common symptoms on presentation were cough, shortness of breath, and vomiting. Each of these symptoms was seen in 71% of patients (n = 5), although not always together. Sinus tachycardia was noticed in 100% of patients (n = 7) and tachypnea in 85% (n = 6). While 85% (n = 6) required hospitalization for respiratory support, all patients were later discharged home on room air. After the diagnosis of EVALI, 85% of patients (n = 6) were treated with steroids.

**Conclusion:**

EVALI is a new disease with unclear mechanisms that commonly presents with symptoms of cough, shortness of breath, and vomiting. It causes severe respiratory compromise in the adolescent population, requiring hospitalization and respiratory support.

## INTRODUCTION

Electronic cigarettes (e-cigarettes) and vaping products are new devices for inhaling various substances such as nicotine and cannabinoids, with or without flavoring chemicals. “Vaping,” or “Juuling,” is a term used to describe the use of e-cigarettes and vaping products.^1^ These devices, also known as e-cigs, vape pens, vapes, mods, pod-mods, tanks and electronic nicotine delivery systems, are available in different shapes and sizes.^1^,^2^

All e-cigarettes and vaping products are made of three components. The first component is the cartridge that contains e-liquid and the atomizer, a coil that heats and converts e-liquid into aerosols. E-liquids can be broadly categorized into two types: regular e-liquids made of propylene glycol-containing chemical flavors and vegetable glycerine used to dissolve nicotine or cannabis e-liquids containing tetrahydrocannabinol and cannabidiol. The second component is the sensor that activates the coil, and the third component is the battery.^1^,^3^ The hookah, also known as a water pipe, is an ancient method of smoking nicotine. In this method, the coal heats the tobacco and then the smoke passes through the water reservoir before it is inhaled.^4^ Contrary to public perception, hookah use is also associated with oral, lung, and esophageal cancers, similar to smoking cigarettes.^4^ In our study, we focused on e-cigarettes, and vaping, product-use associated lung injuries (EVALI).

According to the United States Centers for Disease Control and Prevention (CDC), in 2018 e-cigarettes were used by 3.05 million high school and 570,000 middle school students.^5^ EVALI is a diagnosis of exclusion, with a definition outlined by the CDC for confirmed and probable cases.^6^ EVALI was first identified in August 2019 after the Wisconsin Department of Health Services and the Illinois Department of Public Health received multiple reports of a pulmonary disease of unclear etiology, possibly associated with the use of e-cigarettes and related products.^6^ Since then, more than 2000 cases of EVALI have been reported, and in 80% tetrahydrocannabinol (THC)-containing products were used.^7^,^8^ Our study aimed to identify the clinical characteristics and hospital course of adolescents diagnosed with EVALI.

## CASE SERIES

### Methods

We performed a retrospective chart review of adolescents (11–18 years old) presenting to our hospital between January–December 2019, with diagnosis of EVALI. Subjects were identified by the *International Classification of Diseases, Tenth Revision* (ICD-10) diagnostic codes outlined by official ICD-10 guidelines.^9^ The following codes were used: J68.0 (Bronchitis and pneumonitis due to chemicals, gases, fumes and vapors; includes chemical pneumonitis); J69.19 (Pneumonitis due to inhalation of oils and essences; includes lipoid pneumonia); J80 (Acute respiratory distress syndrome); J82 (Pulmonary eosinophilia, not elsewhere classified); J84.114 (Acute interstitial pneumonitis); J84.89 (Other specified interstitial pulmonary disease); J68.9 (Unspecified respiratory condition due to chemicals, gases, fumes, and vapors); T65.291 (Toxic effect of other nicotine and tobacco, accidental); and T40.7X1 (Poisoning by cannabis or its derivatives, accidental).

We used a standardized data collection sheet. Data were collected by trained personnel who were not blinded to the objectives of study. The data extracted from the medical records were age, gender, weight, and vital signs (temperature, respiratory rate, blood pressure, heart rate) obtained in the ED. We also compiled data on duration of symptoms, history of cough, shortness of breath, chest pain, vomiting, wheezing, rales, use of accessory muscles, and presence of altered mental status. We also included data on respiratory support, duration of hospital stay, use of steroids during treatment, and laboratory tests and imaging obtained in the hospital (complete blood count, respiratory virus panel, respiratory cultures, computed tomography [CT] findings.

We identified cases using the CDC definition of adolescents who had used e-cigarettes or other vaping devices in the 90 days prior to presentation, with bilateral airspace disease on chest imaging (radiograph and/or CT) and a negative infectious workup or the decision by the clinical care team to treat as a case of EVALI.^6^ Exclusion criteria were gastrointestinal and central nervous system (CNS) manifestations without interstitial pulmonary involvement, ingestions of cannabinoids, duplicate visits, and if it was unclear whether vaping device was used or not. We used descriptive statistics to analyze the data. Median and interquartile range (IQR) were calculated for continuous variables, and proportions were calculated with 95% confidence intervals (CI) for categorical variables. The study was approved by the Loma Linda University Institutional Review Board.

**CPC-EM Capsule**

What do we already know about this clinical entity?

Electronic cigarettes (e-cigarettes), and vaping, product-use associated lung injuries (EVALI) is a growing disease entity, especially in adolescents, with diagnostic criteria outlined by Centers for Disease Control and Prevention (CDC).

What makes this presentation of disease reportable?

In our series, we aim to increase awareness by highlighting the clinical presentation of EVALI in the emergency department and its outcome.

What is the major learning point?

EVALI often presents with respiratory and gastrointestinal symptoms, leading to acute respiratory failure, requiring hospital admissions.

How might this improve emergency medicine practice?

*Asking a single question about the use of E-cigarette and vaping products can help diagnose the condition in the context of diagnostic criteria outlined by CDC, differentiating it from other respiratory ailments.*

## RESULTS

We identified 16 encounters with the ICD-10 codes for EVALI during the one-year period. Using the exclusion criteria mentioned in the Methods section, we excluded seven patients. Four of these patients presented with CNS manifestations (seizure or altered mental status) and vomiting without pulmonary involvement. In one patient, the history of vaping was unclear. One patient had ingested cannabinoids without vaping. Two encounters were excluded because they were duplicate visits.

Of the seven patients included in the analysis, six (85%) were male. The median age was 16 years (IQR 15–16). The median weight in our series was 70 kilograms (IQR 63–84). The medians for vital signs recorded in the ED were the following: temperature of 100.2º Fahrenheit (IQR 98.6–102.6); respiratory rate 24 breaths per minute (IQR 18–32); oxygen saturation, 90% (IQR 87–96); heart rate 130 beats per minute (IQR 118–143); systolic blood pressure 128 millimeters of mercury (mm Hg) (IQR 112–139); and diastolic blood pressure 76 mm HG (IQR 64–79). Three (43%; 95% CI, 9–81) patients had documented fever in the ED.

The most common symptoms reported in our study were cough, shortness of breath, and vomiting, each occurring separately in five patients. Three patients presented with chest pain. Two patients presented with altered mental status in the form of unresponsiveness, with one patient requiring intubation. The other unresponsive patient, a 16-year-old male, returned to a normal mentation with bag-valve-mask ventilation and naloxone but required high-flow nasal cannula (HFNC) for shortness of breath. On physical examination, accessory muscle use was the most common finding, reported in four patients. Rales were appreciated in two patients, while no patients were found to have wheezing (Table 1).

In our study, six patients (86%; 95% CI, 42–99) presented with respiratory failure. Four (57%; 95% CI, 22–96) required HFNC. One patient (14%; 95% CI, 0–57) was intubated; one patient (14%; 95% CI, 0–57) required simple nasal cannula oxygen at two liters per minute; and one patient (14%; 95% CI, 0–57) maintained normal oxygen saturations in room air during his ED visit and was discharged home. A brief clinical presentation, summary of findings on imaging, and type of respiratory support needed are summarized in Table 2.

Five patients (71%; 95% CI, 36–99) were admitted to the pediatric intensive care unit, and one patient (14%; 95% CI, 0–57) was admitted to the normal pediatric unit. The median hospital length of stay was six days (IQR 3–7). All patients (100%) were discharged with no comorbidities or deaths reported. Six patients (86%; 95% CI, 42–99) were treated with steroids. The median duration of treatment with steroids during admission and after discharge was nine days (IQR 5–10).

Our patients had a variety of laboratory tests ordered. Most common were complete blood count, respiratory virus panel, respiratory cultures, and urine drug screen. All patients had a complete blood count, and the median for white cell count was 16 thousand cells per cubic millimeter (reference range 4.80–11.80; IQR 11–21). A respiratory virus panel was collected from five patients and it was negative in all of them (0%; 95% CI, 0–50). Respiratory cultures were collected from two patients and both resulted negative. A urine drug screen was performed for six patients and was positive for cannabinoids in all six (100%; 95% CI, 63–100).

Three patients (43%) followed up at different intervals in the pulmonology clinic (Table 1: Cases 1, 2 and 4). Spirometry showed normal results in all three patients (100%) at that time. Case 1 followed up one week after discharge, at which time spirometry showed evidence of obstructive lung disease, which returned to normal at three-month follow-up visit. No repeat imaging was performed for that patient. Case 2 followed up six weeks after discharge with near-complete resolution of ground-glass appearance on repeat CT and normal spirometry. Case 4 followed up two weeks after discharge with improvement in lung opacities on repeat radiograph and normal spirometry. All three patients had received steroids for 10 days when they were originally diagnosed with EVALI. No follow-up data was available for the remaining four patients.

## DISCUSSION

EVALI was an emerging disease entity in 2019. In our case series, we describe adolescents diagnosed with EVALI and their clinical course in the ED and the hospital. In our study, the most common symptoms of cough, shortness of breath, and vomiting presented with an equal frequency of 71%. In a study by Layden et al, shortness of breath and cough was noticed in 85% of patients and vomiting in 61%; whereas, according to Belgaev et al, 90% of patients in their study presented with gastrointestinal (GI) and respiratory symptoms.^6^,^10^ In a report by the CDC, 85% of the EVALI population had respiratory symptoms and 57% had GI symptoms.^11^ The results of our study are similar to previous literature in suggesting that respiratory and GI symptoms are common in patients with EVALI.

According to Balgaev et al, 67% of patients had clinical and radiological improvement with residual findings on radiological and pulmonary function tests at time of follow-up. 10 In our study, the three patients who had documented follow-up visits had normal spirometry without residual deficits. Only two of those patients had repeat imaging, and both showed improvement without residual abnormalities.

E-cigarette liquids and aerosols have been shown to contain a variety of chemical constituents including flavors that can be cytotoxic to human pulmonary fibroblasts and stem cells.^12^ Exposure to heavy metals such as chromium, nickel, and lead has also been reported. 13 None of our patients were tested for heavy metal exposure. Most of the delivery systems have nicotine in them, with one cartridge providing the nicotine equivalent to a pack of cigarettes.^12^

In addition to nicotine, e-cigarette devices can be used to deliver THC-based oils.^14^ According to Trivers et al, one-third of the adolescents who used e-cigarettes had used cannabinoids in their e-cigarettes. 15 In our patients with EVALI, urinary drug screen was positive for cannabinoids in all patients. One caveat is that we do not know whether our patients used only THC-containing products or a combination of nicotine and THC-containing products.

In our case series, the majority of patients presented with pulmonary disease requiring respiratory support and intensive care unit admission. None of these patients developed acute respiratory distress syndrome (ARDS). We likely did not see this disease process due to our small sample size, as Layden et al reported ARDS development in several of their examined cases.^6^ In our series, we did not evaluate the pathologic pulmonary changes in different patients. In other case reports, different pathophysiologic patterns of pulmonary involvement, in the form of diffuse alveolar hemorrhage, exogenous lipoid pneumonia, acute eosinophilic pneumonia, or hypersensitivity pneumonitis have been identified.^16^–^19^

Although the mechanism of EVALI is not clearly understood, the CDC suggests the use of steroids for treatment.^11^ According to a series of patients in Illinois, 51% of those patients had improvement in symptoms after the administration of steroids.^6^ In another study, patients showed clinical and radiological improvement following the use of antibiotics and steroids.^10^ In our study, six patients received steroids and six patients received antibiotics; three of those patients followed up in clinics with normal spirometry. But this evidence is not sufficient to establish that use of steroids or antibiotics is beneficial in EVALI.

There are several limitations of our study. First, because it was a retrospective chart review we could not establish causation. Second, all data may not have been recorded on all patients (such as smoking THC vs nicotine vs both). We might have missed some if the ICD-10 codes were not correct on the chart. Only three had documented follow-up, so we don’t know whether the other four had any comorbidities after their hospitalization. Third, we had a small number of patients. Fourth, this was a single-center study; so results may not be generalizable to other hospitals with different patient demographics.

## CONCLUSION

Our study supports that EVALI can present with respiratory and gastrointestinal symptoms. It can lead to acute respiratory failure requiring respiratory support and hospital admission. Respiratory complaints are a common reason for adolescents to present to the ED, and physicians should consider EVALI when there is a history of recent use of vaping devices. We also suggest counseling adolescents against use of e-cigarette with and without THC-containing products, whenever that history is elicited in the ED.

## Figures and Tables

**Table 1:** Common presenting signs and symptoms of EVALI: electronic cigarettes, and vaping, product-use associated lung injury.

Signs and symptoms	N (percentage; 95% CI)
Cough	5 (71%; 36–99)
Shortness of breath	5 (71%; 36–99)
Vomiting	5 (71%; 36–99)
Chest pain	3 (43%; 9–81)
Accessory muscle use	4 (57%; 22–96)
Tachycardia	7 (100%; 63–100)
Tachypnea	6 (85%; 42–99)
Rales	2 (29%; 5–85)
Altered mental status	2 (29%; 5–85)

*N*, total number; *CI*, confidence interval.

**Table 2:** Characteristics of adolescents with electronic cigarettes, and vaping, product-use associated lung injury.

Cases	Age (yrs)	Gender	Clinical presentation	Imaging (CT)	Respiratory support	Steroid duration (Days)	Antibiotics	Disposition	Length of stay in hospital (Days)
1	17	M	Fever, shortness of breath and cough for 3 days with 5 episodes of non-bloody, non-bilious vomiting	Bilateral, lower lobe predominant ground glass attenuation with subpleural sparing	HFNC 20 lpm FiO_2_ 40%	10	Yes	PICU	6
2	16	F	History of epilepsy. Had a self-resolved seizure followed by development of a cough, shortness of breath, chest pain and hemoptysis	Bilateral ground glass opacities with interlobular septal thickening predominantly in the mid and upper lung zones	HFNC 20 lpm FiO_2_ 100%	10	Yes	PICU	4
3	16	M	Found unresponsive, locked in his room. Arousable but developed acute respiratory failure and required intubation	Right pneumothorax, dense consolidation of right lung/partial white out, L lung base consolidation, subcutaneous emphysema	Intubated ACPC 26/5 FiO_2_ 40%	0	Yes	PICU	10
4	16	M	Non-productive, dry cough for 5 days followed by worsening shortness of breath for 1 day	Extensive bilateral patchy areas of infiltration with some basilar consolidation and small bilateral pleural effusions	Nasal Cannula 2 lpm	10	Yes	Pediatric in-patient unit	7
5	15	M	Vaping marijuana and snorting acetaminophen-oxycodone; was found unresponsive by family. Returned to normal mentation with naloxone, but developed hypoxia, chest pain and shortness of breath	Fluffy centrilobular opacities in central and peripheral lungs, with prominence at posterior lower lobes	HFNC 10 lpm FiO_2_ 35%	5	No	PICU	3
6	15	M	Vomiting once per day for 5 days followed by cough and chest pain for 2 days	Reticular opacities, particularly in bi-basilar subpleural space, may represent mild/early fibrosis but chronicity has not been established	Room air	5	Yes	Discharged home	N/A
7	16	M	Abdominal pain and diarrhea for 2 weeks, followed by non-bloody, non-bilious vomiting and shortness of breath for 2 days	Mild granular and patchy parenchymal opacities in both lung bases concerning for bilateral pneumonia	HFNC 20 Lpm FiO_2_ 21%	9	Yes	PICU	7

*CT*, computed tomography; *HFNC*, high-flow nasal cannula; *lpm*, liters per minute; *PICU*, pediatric intensive care unit; *ACPC*, assist control, pressure-controlled setting; *FiO_2_*, fraction of inspired oxygen.
